# The mCME Project: A Randomized Controlled Trial of an SMS-Based Continuing Medical Education Intervention for Improving Medical Knowledge among Vietnamese Community Based Physicians’ Assistants

**DOI:** 10.1371/journal.pone.0166293

**Published:** 2016-11-18

**Authors:** Christopher J. Gill, Bao Le Ngoc, Nafisa Halim, Ha Nguyen Viet, Anna Larson Williams, Tan Nguyen Van, Marion McNabb, Lien Tran Thi Ngoc, Ariel Falconer, Hai An Phan Ha, Julia Rohr, Hai Hoang, James Michiel, Tam Nguyen Thi Thanh, Liat Bird, Hoang Pham Vu, Mahlet Yeshitla, Nhu Ha Van, Lora Sabin

**Affiliations:** 1 Center for Global Health and Development, Boston University, Boston, MA, United States of America; 2 Department of Global Health, Boston University School of Public Health, Boston, MA, United States of America; 3 Pathfinder International in Vietnam, Hanoi, Vietnam; 4 Center for Population Research Information and Databases (CPRID), Ministry of Health, Hanoi, Vietnam; 5 General Office for Population and Family Planning (GOPFP), Ministry of Health, Hanoi, Vietnam; 6 Pathfinder International, Watertown, MA, United States of America; 7 Thái Nguyên Provincial Department of Public Health, Thái Nguyên City, Vietnam; 8 Hanoi Medical University, Hanoi, Vietnam; 9 Boston University School of Medicine, Boston, MA, United States of America; 10 Hanoi School of Public Health, Hanoi, Vietnam; University of South Australia, AUSTRALIA

## Abstract

**Background:**

Community health workers (CHWs) provide critical services to underserved populations in low and middle-income countries, but maintaining CHW’s clinical knowledge through formal continuing medical education (CME) activities is challenging and rarely occurs. We tested whether a Short Message Service (SMS)-based mobile CME (mCME) intervention could improve medical knowledge among a cadre of Vietnamese CHWs (Community Based Physician’s Assistants–CBPAs) who are the leading providers of primary medical care for rural underserved populations.

**Methods:**

The mCME Project was a three arm randomized controlled trial. Group 1 served as controls while Groups 2 and 3 experienced two models of the mCME intervention. Group 2 (passive model) participants received a daily SMS bullet point, and were required to reply to the text to acknowledge receipt; Group 3 (interactive model) participants received an SMS in multiple choice question format addressing the same thematic area as Group 2, entering an answer (A, B, C or D) in their response. The server provided feedback immediately informing the participant whether the answer was correct. Effectiveness was based on standardized examination scores measured at baseline and endline (six months later). Secondary outcomes included job satisfaction and self-efficacy.

**Results:**

638 CBPAs were enrolled, randomized, and tested at baseline, with 592 returning at endline (93.7%). Baseline scores were similar across all three groups. Over the next six months, participation of Groups 2 and 3 remained high; they responded to >75% of messages. Group 3 participants answered 43% of the daily SMS questions correctly, but their performance did not improve over time. At endline, the CBPAs reported high satisfaction with the mCME intervention, and deemed the SMS messages highly relevant. However, endline exam scores did not increase over baseline, and did not differ between the three groups. Job satisfaction and self-efficacy scores also did not improve. Average times spent on self-study per week did not increase, and the kinds of knowledge resources used by the CBPAs did not differ between the three groups; textbooks, while widely available, were seldom used.

**Conclusions:**

The SMS-based mCME intervention, while feasible and acceptable, did not result in increased medical knowledge. We hypothesize that this was because the intervention failed to stimulate lateral learning. For an intervention of this kind to be effective, it will be essential to find more effective ways to couple SMS as a stimulus to promote increased self-study behaviors.

**Trial Registration:**

ClinicalTrials.gov NCT02381743

## Introduction

Globally, community health workers (CHWs) are a key source of basic health care to hundreds of millions of people.[1–4] In fact, in many low-resource countries, CHWs are the only source of health care. Because CHWs’ ability to deliver quality services is dependent on their level of professional knowledge,[5, 6] developing ways to support this is critical. New technologies, including mHealth approaches, offer affordable opportunities for distance learning at a scale never before possible.

In the US and many other high-income countries, clinicians are required to maintain clinical knowledge through participation in yearly, accredited CME activities. Increasingly, these are delivered via internet-based, self-educational modules, such as a clinical vignette accompanied by multiple-choice questions. Correct answers and CME credits are awarded to the participant upon satisfactory completion of the module. This is the strategy used by some professional medical societies for their maintenance of certification activities, on-line medical information vendors (such as Medscape), and many medical publications, including JAMA and the British Medical Journal. In each of these examples, the learner completes his/her work using a personal computer, tablet computer or a smart phone. However, this approach could be adapted for cell phones using SMS, an advantage in settings where cell phone ownership is ubiquitous but access to the Internet is limited.

As with many countries, the Government of Vietnam is seeking to develop strategies to maintain the skills of its medical workforce. In November 2009, Vietnam passed the ‘Law on Medical Examination and Treatment’ mandating that all clinicians be licensed and that CME be required to sustain licensure. This applies not just to doctors and nurses, but also to mid-level providers, including a pivotal cadre of CHW called ‘Community Based Physician’s Assistants’ (CBPAs). CBPAs are the main source of primary care in Vietnam, and often the only local source for rural and underserved populations. Despite their title, CBPAs often work without direct oversight from a physician.

Given limited resources, providing CME opportunities to CBPAs is proving to be a formidable challenge. It also presents an opportunity to test innovative and low cost strategies for delivering CME using distance learning. In the mobile Continuing Medical Education Project (mCME Project), we conducted a randomized controlled trial to test whether an SMS-based CME intervention could improve the medical knowledge of CBPAs. Secondary objectives included the impact of the intervention on CBPA’s job satisfaction and perceived self-efficacy.

## Methods

### Study overview

Study participants were practicing CBPAs living and working in Thái Nguyên Province, a rural agricultural region north of Hanoi. All CBPAs had completed secondary school, had graduated from an accredited 2-year medical training college program, and were active clinicians working in primary care at provincial Commune Health Centers. Study inclusion required ownership of an SMS-enabled cell phone (smart phones or feature phones were both acceptable), ability to receive SMS at their place of work, being 18 years of age or above, and being willing to participate in all study activities. Ethical oversight was provided by the institutional review boards at Boston Medical Center and The Hanoi School of Public Health. The study was registered on clinicaltrials.gov as NCT02381743. All subjects provided written informed consent. The protocol can be accessed at **S1 Protocol**.

The mCME project was designed as a three-arm randomized controlled trial, with efficacy assessed by a pair of medical knowledge examinations administered at baseline (time zero) and endline (six months later). Group 1 served as controls: these individuals received a weekly non-medical SMS message, with no response back to the server required. Groups 2 and 3 were both intervention groups. In Group 2, participants received a daily medical fact, or bullet point, addressing some aspect of primary care medicine. Upon receipt, Group 2 participants were instructed to reply using any combination of numbers or characters to acknowledge receipt.

**Fig 1:**
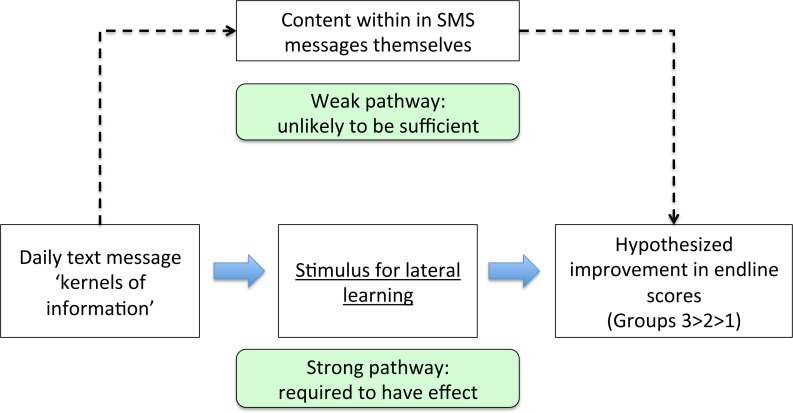
Conceptual model for how the mCME intervention was hypothesized to work to improve medical knowledge. The figure outlines our model for the mCME intervention. The figure outlines two pathways that could lead to our desired output: improvement on the endline exam score. The first is learning from the information within the SMS messages themselves (the weak pathway). However, these are very brief, cover each topic superficially, and so would not be expected to have much effect per se. Rather, the SMS was hoped to serve as a stimulus promoting increased self-study on the same thematic areas as addressed in the messages through lateral learning (the strong pathway).

**Fig 1**lays out the conceptual model for how the mCME intervention was hypothesized to work to improve medical knowledge among CBPAs. Pedagogical research shows that knowledge retention is improved when content is delivered in an interactive fashion.[7–10] Conversely, merely providing individuals with information may be ineffective.[10] Anticipating that daily ‘bullet points’ could become tiresome or even viewed as spam, Group 3 participants received a daily message covering the same thematic area as Group 2, but instead formatted as a four-option multiple choice question. For example, a daily message to Group 2 could be, “*Danger signs of pregnancy include swelling ankles*, *vaginal bleeding*, *headaches and fever*”, whereas the Group 3 version might be, “*Which of the following is a danger sign of pregnancy? A*. *Abdominal bloating; B*. *Early satiety; C*. *Fever; D*. *Easy fatigability*”, with item ‘C’ being the correct answer.

The SMS system was programmed to collate all answers longitudinally by individual participant cell phone numbers (which were linked to study ID numbers), recording when messages were sent, and if and when user responses were received. In the case of Group 3, the system had limited capacity to interpret the responses and provide a counter-response. Extending the example above, these would be either “*Congratulations you’re correct*, *the answer was C*”, or “*I’m sorry*, *the answer was C*.” Responses were interpretable if in the format: ‘a’, ‘b’, ‘c’, ‘d’, or ‘A’, ‘B’, ‘C’, ‘D’. All other responses were non-conforming, and triggered the corrective response from the server, “*I’m sorry*, *the answer was [X]*.”

We did not use a sampling strategy to select CBPAs based on pre-defined criteria. Rather we sought to enroll as many of the CBPAs in Thái Nguyên province as we could. All of these CBPAs practicing were invited by mail by the Thái Nguyên Department of Public Health to join the study. Since we could not know in advance how many would respond to the invitations, we had prepared sufficient ID numbers and test materials to accommodate beyond our 660 subject target. CBPAs were invited to attend one of six sessions held over three consecutive days in May 2015 at the Thái Nguyên Department of Public Health in Thái Nguyên City. The endline evaluations were held at the same location six months later in November 2015.

After the project was explained to participants who presented at the workshops, questions were solicited and addressed, and consent forms signed. Consenting participants queued up at one of five registration stations, and were then assigned a unique study ID number, which was then linked to the participant’s cell phone number. In advance of the workshops, the study statistician used a random number generator to allocate the study ID numbers in a 1:1:1 ratio to the three study groups using block randomization. Each ID number was pre-assigned to specific versions and sub-versions of the baseline and endline examinations (see below). Subjects were assigned ID numbers as they enrolled sequentially. A test SMS was sent immediately from the server to each participant to confirm that the phone number was correct and the phone functioning. At the baseline workshop, held in May 2015, participants completed a series of demographic questions, along with questionnaires assessing job satisfaction and self-efficacy using two validated tools, the Brief Index of Affective Job Satisfaction (BIAJS) and the Core Self Evaluation (CSE) tools, respectively.[11, 12] They then took a timed, 100-item, 90-minute medical examination. The intervention commenced the following Monday morning, with weekly (Group 1) or daily (Groups 2 and 3) SMS being sent out in batches every morning over the next six months.

At the end of six months, participants were then asked to return for the endline evaluation held over six half-day sessions, where they again completed the CSE and BIAJS questionnaires, answered questions on self-study habits, and then took the second timed, 100-item, 90-minute examination. This endline examination covered exactly the same thematic areas as the first exam, but did not repeat any of the questions.

### Development of the SMS delivery software

The software for delivering the SMS messages was developed for the study by the information technology team at the Center for Population Research Information and Databases (CPRID), a sub-division of the Global Office for Population and Family Planning (GOPFP) within the Vietnamese Ministry of Health (MOH). By developing the software in-house on MOH servers, the Vietnamese government had ownership of the requisite technology to facilitate future use. The system used a GSM modem to pull the daily messages from group-linked content data files, and to send these out in batches by study Group starting at 9 AM each morning, seven days a week. The system collated responses by time and ID number, and for Group 3, whether participant answers were correct or not. Response rates were tabulated to allow us to refund participants for all talk time minutes expended in replying to the SMS messages.

### Development of exam and SMS content sets

SMS and exam content was developed by the research team, based on primary care syllabi from the Thái Nguyên Medical College’s CBPA curriculum. We created 180 content sets distributed across six topic areas (surgery, internal medicine, pediatrics, infectious diseases, sexually transmitted diseases, and family planning), with 20–40 thematically-linked content sets in each area. Each final message was retrofitted into 160 characters or less. For example, a content set for an internal medicine question under the theme Type II Diabetes would contain: 1) two examination questions with corresponding answers; 2) a Group 2 non-question SMS; and 3) a Group 3 SMS question and answer. Themes were often used more than once, but none of the content items was ever repeated. A team of six medical and public health students at Boston University developed the initial content sets, which were reviewed and edited by the principle investigator. Each set was then revised, approved, and validated over a multi-cycle iterative process between the Boston-based team and colleagues at the Hanoi Medical University and Hanoi School of Public Health. Because each SMS had a 160-character limit, and the additional phonetic marks in Vietnamese script counted against this limit, these marks were omitted following pilot testing with CBPAs to confirm they could understand messages without the marks.

Group 3 SMS questions were ‘first order’ questions, meaning that they solicited a simple answer to a direct question, such as ‘*What is the optimal antibiotic for streptococcal pharyngitis*?’ (Answer: Amoxicillin). By necessity, these had to be brief to avoid exceeding the 160-character limit. The examination questions, by contrast, were ‘second’ or ‘third order’ complexity, meaning that they required integration of two or more separate pieces of medical knowledge to answer. For example, “*A child with a sore throat developed a macular rash after taking an antibiotic*. *What was the most likely cause of the sore throat?”* (Answer, mononucleosis.) Answering correctly required a CBPA to know the differential diagnosis of sore throats, the most likely treatment (amoxicillin) for streptococcal pharyngitis, and the fact that macular rash is a common result of using amoxicillin in the setting of mononucleosis.

Not all themes covered by the SMS were assessed in the examinations but all themes covered in the examinations were associated with corresponding SMS messages. The baseline and endline examinations were intended to be challenging to avoid a ceiling effect.

To balance the difficulty of the baseline and endline examinations, we treated this as a statistical confounder, and resolved it by randomly selecting which of the two exam questions within a given content set would end up in the baseline or endline versions. Because the baseline and endline examinations covered the same topics but did NOT include the same questions, this meant that the exams were designed to test for the acquisition of new knowledge, and were not tests of memory of the prior exam. Rather, the paired examinations were constructed to be equally difficult (making use of randomization to select among the question pairs), but different from each other. This is the same general approach used for common standardized tests in the US, such as the Scholastic Aptitude Test, Medical College Admissions Test, and others: students can take these repeatedly, and the exams are designed to be equally challenging. But the questions do not repeat, ensuring that every time will be the first time a test taker encounters a given question.

### Examination procedures

At baseline and endline, each participant was given a sealed examination package. To avoid transcription errors, each packet was pre-filled on the cover with the subject’s unique ID number. Inside the package was a bubble sheet, also pre-filled with the subject’s unique ID number, and the survey and examination materials. Also, participants were provided with a reminder card (specific to each group) summarizing the intervention and what they were expected to do, and providing them with a hot line number to call in the event of questions. Participants were walked through the demographic and survey questions as a group to ensure clarity regarding questions and completion of items. Once completed, the examination began.

**Fig 2:**
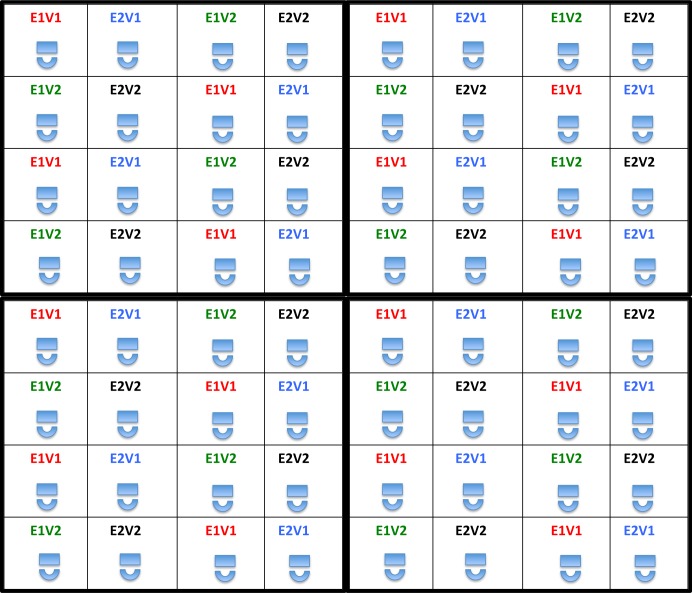
Seating diagram for baseline and endline evaluations. Each cell represents an individual sitting a desk taking a specified version and sub-version of the examination. Each 4x4 block of sixteen seats can be repeated ad infinitum, and will never result in two individuals who are taking the same exam versions sitting adjacent to each other in any direction. This was intended to minimize potential that participants could work together or share answers during the baseline and endline evaluations. It can flexibly be adapted to suit different sized/shaped rooms. **E1V1** = Exam version 1, sub-version 1; **E1V2** = Exam version 1, sub-version 2. **E2V1** = Exam version 2, sub-version 1; **E2V2** = Exam version 2, sub-version 2.

Examinations were proctored with study team members walking through and observing the room at all times. To further reduce the risk of sharing answers or working collectively, within each main exam version, we created two sub-versions by randomly shuffling the sequence of questions. In this way, all participants were presented with both sets of exam questions, either at baseline or endline, but with the questions occurring in two different sequences. These four exam versions (v1.1, v1.2 and v2.1, v2.2) were linked to study ID numbers to ensure that each participant took both main versions of the exam during the study. A seating grid was used to ensure that nobody was seated next to another taking the same version of an exam at the same time (**Fig 2**). The cover of each examination package was printed with a number (1–4), which corresponded to a seat in the examination room, all of which were also labeled 1–4, corresponding to the four different versions of the exam. Participants were instructed to find a seat that matched the number on their exam packet’s cover.

The examination materials were retrieved at the end of each session, and the bubble sheet data scored automatically using an optical mark scanner with essentially 100% fidelity.

### Sample size considerations

The study was powered to detect a 10% difference in mean group scores between each of the three groups. Our hypothesis was that Groups 2 and 3 would outperform Group 1 on the endline examination, and that the interactive model (Group 3) would outperform the passive model (Group 2). For this calculation, we assumed a continuous response variable from independent controls and experimental subjects with 1:1 matching for each pairwise comparison. Since we had never administered the examination before, we estimated a standard deviation that was 3.5 times larger than the desired measurable delta of 10%, for an SD of 35%. With those parameters, a two-sided t-test, an alpha of 0.05 and beta of 0.8, we estimated that 193 individuals per group would be sufficient. We rounded that up to 200 and then applied a 10% attrition rate after the baseline exam, which resulted in the final sample size of 220 per group.

### Statistical analysis

SAS version 9.4 was used for all analyses. Our primary endpoint was a comparison of average endline test scores, using paired t-tests and ANOVA as required. Secondary comparisons of the CSE and BIAJS scores required converting the individual item responses on both tools into composite scores, which were also compared using t-tests and ANOVA as required. Non-parametric tests were used to contrast dichotomous or multi-level responses, such as hours spent in formal medical self-education at baseline/endline.

To evaluate factors explaining differences in scores, we constructed three sets of linear regression models. The first used ‘endline evaluation mean score’ as the dependent variable, controlling for group allocation, gender, age, years worked as CBPA, and other baseline demographics. The second model was restricted to Group 2 and 3 participants, and included the additional explanatory variable ‘*Average response rate’* to the daily messages. The third model was restricted to Group 3 participants, and included the additional explanatory variable ‘*Correct answer rate*’ (to the daily questions). Additional descriptive and summary statistics were generated to compare response rates between Groups 2 and 3 over time, and across other stratifications. The final data sets can be accessed on line at **S1 Data**.

## Results

### Cohort profile

**Fig 3:**
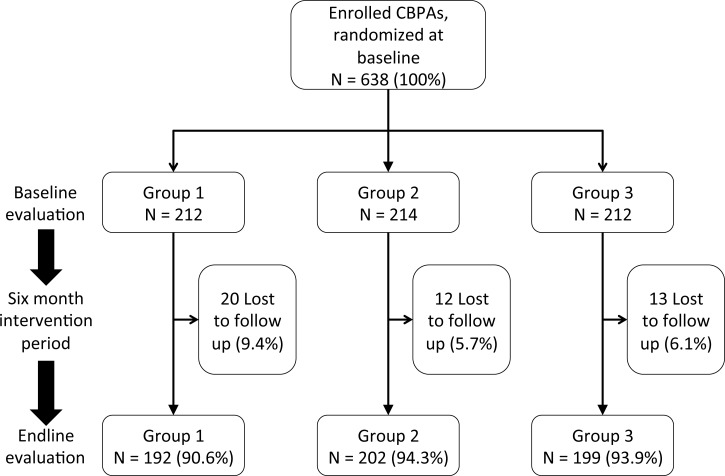
Study flow diagram.

The study was conducted between May and November 2015, and completed once the endline workshops had been conducted. At baseline, 638 (96.7% of our target sample size) subjects were enrolled and randomized. Of these, 593 returned for the endline evaluation, with 7.1% attrition due to loss to follow up (**Fig 3**). Attrition was similar in Groups 2 and 3 (P = NS), and somewhat higher in Group 1 (P = 0.1 in Group 1 vs. 2 and Group 1 vs. 3 comparisons), but was <10% in all Groups. All CBPAs who came to the baseline workshops consented to the study, and none withdrew consent subsequently.

Demographic characteristics were generally well balanced at baseline between Groups 1–3 (**Table 1**). Overall, participants were predominantly female, and worked in small groups at rural health centers. Less than half reported attending a formal CME training within the preceding two years, and the majority reported spending only 1–2 hours per week on medical self-study. Despite this, >90% reported that their current exposure to CME was sufficient to maintain their clinical skills.

**Table 1:** Characteristics of study participants.

	Group 1 (N = 212)	Group 2 (N = 214)	Group 3 (N = 212)	*P-value*
Gender (% female)	67.92	70.56	67.92	NS
Mean age (Years)	37.65	38.21	37.63	NS
Practice setting (%)				
Rural clinic	72.17	79.44	75.94	NS
Town clinic	18.40	9.81	14.62	≤0.05
City clinic	8.96	10.75	8.49	NS
Patients seen per day (%)				
0–9 patients	38.68	27.57	41.04	≤0.01
10–19 patients	38.68	51.87	36.79	≤0.01
20–29 patients	12.74	11.68	11.79	NS
30–39 patients	6.13	5.61	5.66	NS
40+ patients	2.83	1.87	4.25	NS
No. of clinicians in practice group (including participant) (%)				
1–2	21.23	19.16	18.40	NS
3–4	58.96	62.15	63.68	NS
5–7	12.74	14.02	12.74	NS
8–11	1.89	1.87	2.36	NS
12+	4.72	2.34	1.89	NS
Medical Specialty				
General medicine	58.02	56.07	56.13	NS
Obstetrics & pediatrics	19.34	21.50	19.34	NS
Traditional medicine	12.26	16.82	15.57	NS
Preventative medicine	9.91	5.61	8.02	NS
Attended government sponsored CME training in past 2 years (%)	47.17	45.79	44.34	NS
Hours spent per week on medical self-education				
0 hours	5.19	3.74	4.72	NS
1–2 hours	48.58	51.87	42.92	NS
2–4 hours	23.58	23.83	26.89	NS
4–7 hours	12.26	9.35	14.15	NS
8 or more hours	9.43	11.21	9.91	NS
Reported that they feel that they currently receive sufficient medical training to support their skills (%)	91.04	93.93	87.26	NS

NS = Non-significant

### Interactions between the server and Groups 2 and 3 participants

Out of 180 planned SMS messages (for Groups 2 and 3), the server sent out 177 messages (98.3% success rate). Technical problems with the server accounted for the three missing messages; additional days were not added to the intervention period to compensate.

**Fig 4:**
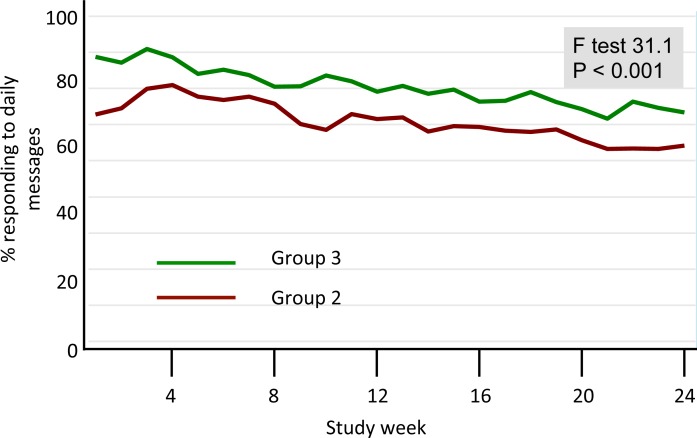
Weekly response rates (Groups 2 and 3). Group 2 and 3 participants were required to respond to the daily SMS messages. The figure summarizes the weekly average response rates for the two groups separately. As can be seen, participation was high for both groups, but statistically significantly higher for Group 3 participants. Group 1 participants were not asked to respond to the messages.

Response rates were high in both intervention groups, though consistently higher in Group 3 than in Group 2 (P<0.001). There was a slow decay in response rates over time for both intervention groups (**Fig 4**). On average, Group 2 participants took approximately 1.5 hours to respond to the daily bullet message, while Group 3 participants took 2.5 hours to reply (P<0.001). Among Group 3 participants, 45% replied to the daily question ≥96% of the time; 35% replied between 84–95% of the time; 10% replied between 72–83% of the time; and the remaining 10% replied to <72% of the messages. Thus, 90% of Group 3 participants answered most or all of the daily questions.

**Fig 5:**
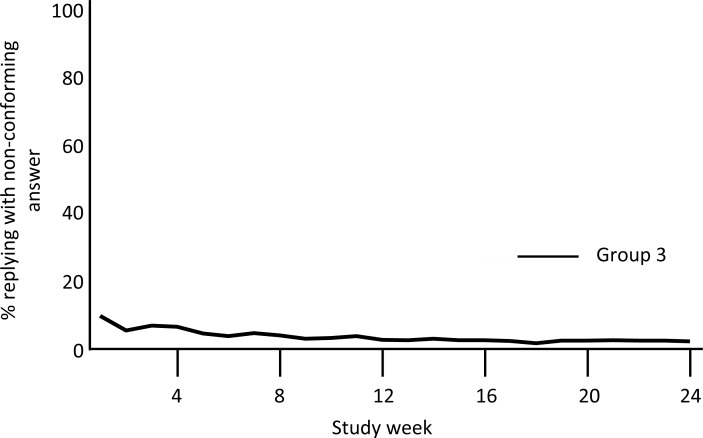
Weekly non-conforming answer rates (Group 3 only). This summarizes the week-by-week average rates of non-conforming answers sent back by Group 3 participants. Responses were interpretable only if in the formats ‘a’, ‘b’, ‘c’, ‘d’, or ‘A’, ‘B’, ‘C’, or ‘D’. All other responses were ‘non-conforming’. Non-conforming answers were most common in the initial few weeks of the study, and then fell to < 10%.

Overall, 85% of answers from Group 3 conformed to the A, B, C, or D format. Non-conforming responses were most common during the first 2 weeks of the intervention, and then fell to <10% of responses for the remainder of the study (**Fig 5**). Further evaluation of these answers revealed several common errors, such as including two or more answers to the daily question (e.g., ‘A, B’), sending emoticons (e.g.,: -X), or writing out the response as a sentence instead of a single letter answer (e.g., ‘*Dap an A’*, translated from Vietnamese as ‘*The answer is A’*). While there was no specific intervention provided in response, such non-conforming responses became less common after the first several weeks of the intervention, suggesting that the subjects were self-correcting based on the replies returned by the server. By contrast, several subjects consistently replied using stereotypical numerical codes, such as ‘0041’, ‘0042’, ‘0043’, and ‘0044’). We hypothesized, and later confirmed, that these were instances where the participant’s phone’s internal settings or operating system created incompatibilities with our software such that ‘letter’ responses were converted into a numeric code. Therefore, these represented programming issues rather than persistent user errors.

**Fig 6:**
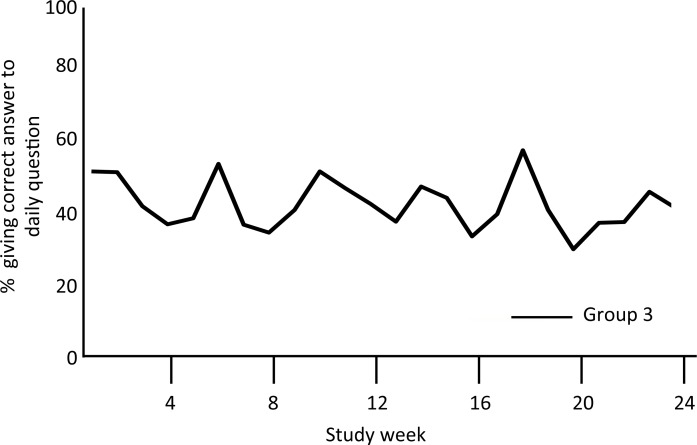
Weekly correct answer rates (Group 3 only). The figure summarizes the proportion of Group 3 participants who provided a correct response to the daily SMS question over the 6 months of the study. While there was considerable week-to-week variation, there was no trend to improvement over time.

Group 3 participants correctly answered 43% of the daily questions, with no improvement over time (**Fig 6**). Random guesses should yield a correct response rate of ~25%.

### Acceptability of the mCME intervention among Group 2 and 3 participants

In response to endline queries about the SMS intervention, the vast majority of Group 2 and 3 participants reported a ‘favorable’ or ‘very favorable’ opinion: 84.7% and 82.4%, respectively. In terms of relevance of SMS content, 93.1% and 93.5% of Group 2 and 3 participants, respectively, reported that the messages were ‘relevant’ or ‘highly relevant’ to their daily clinical work.

### Impact of mCME intervention on primary and secondary study endpoints

At baseline, examination scores were very similar between the three groups, with mean scores within 2.9 percentage points of each other, ranging from 36.1% to 39.0% (P<0.05). As shown in **Table 2**, endline examination scores improved insignificantly (<3 percentage points gain in any group) with virtually no differences between the three groups, either as a comparison of endline mean scores, or mean change in scores from baseline to endline. The scores did not differ between groups when disaggregated by key subject areas (surgery, family planning, internal medicine, pediatrics, infectious diseases, and sexually transmitted diseases–data not shown; available upon request). Similarly, the mCME intervention had no impact on either job satisfaction scores, which were high at baseline and remained high at endline, or perceived self-efficacy scores (**Table 3**).

**Table 2:** Comparison of baseline and endline medical knowledge exam mean test scores, and mean changes in test scores between baseline and endline.

**Group**	**Baseline**^**1**^				
	**n**	**Mean % score**	**std. dev**	**min**	**max**
**1**	212	37.6	9.9	13	63
**2**	214	36.1	9.6	12	56
**3**	212	39.0	9.5	8	57
	**Endline**^**2**^				
**Group**	**n**	**Mean % score**	**std. dev**	**min**	**max**
**1**	192	40.5	10.0	9	60
**2**	202	40.1	9.2	8	62
**3**	199	40.9	10.4	6	67
**Group**	**Mean change in scores**^**3**^				
	**(mean endline minus mean baseline scores)**				
	**n**	**Mean change (% points)**	**std. dev**	**min**	**max**
**1**	192	3.0	8.7	-26	34
**2**	202	4.1	9.7	-18	37
**3**	199	1.7	9.5	-30	28

Notes

1.P value significant at α = .05 for comparison of Groups 1–3 mean scores at baseline

2.P value non-significant for comparison of Groups 1–3 mean scores at endline

3.P value non-significant for comparison of degree of change from baseline to endline between Groups 1–3.

**Table 3:** Impact of mCME intervention on key secondary endpoints: self-efficacy and job satisfaction.

	Group 1		Group 2		Group 3	
Secondary endpoint	Baseline	Endline	Baseline	Endline	Baseline	Endline
**Self Efficacy (Core Self Evaluation tool)**	43.2	43.1	43.1	42.6	42.7	42.2
(Maximum score 60 points)		P = NS		P = NS		P = NS
**Job satisfaction (Brief Index of Affective Job Satisfaction tool)**	35.7	35.5	36.1	36.1	36.1	36.0
(Maximum score 45 points)		P = NS		P = NS		P = NS

### Impact of mCME intervention on participants’ self-study behaviors

The mCME intervention did not lead to a significant change in the number of hours spent each week on formal CME self-education. At baseline and endline, participants reported spending 1–2 hours / week on self-study. When asked at endline about the frequency of using various resources in self-study, there were no significant differences between the three groups in proportions that consulted textbooks and that consulted with colleagues (**Table 4**). However, significantly higher proportions of participants in Groups 2 and 3 reported attempting to locate medical information on the Internet compared to Group 1.

**Table 4:** Impact of mCME intervention on endline self-education behaviors.

	Group 1		Group 2		Group 3	
Used textbooks	Mean	SD	Mean	SD	Mean	SD
Daily	26.6%	44.3%	34.7%	47.7%	30.2%	46.0%
Weekly	41.1%	49.3%	32.2%	46.8%	28.6%	45.3%
Monthly	11.5%	31.9%	11.4%	31.8%	13.1%	33.8%
This resource was not available	13.5%	34.3%	17.3%	37.9%	21.6%	41.3%
Resource available, but not used	6.3%	24.3%	3.0%	17.0%	4.5%	20.8%
**Consulted with colleagues**						
Daily	83.3%	37.4%	86.1%	34.6%	85.4%	35.4%
Weekly	13.5%	34.3%	8.9%	28.6%	8.0%	27.3%
Monthly	0.0%	0.0%	1.0%	9.9%	3.5%	18.5%
This resource was not available	0.5%	7.2%	1.5%	12.1%	0.0%	0.0%
Resource available, but not used	2.1%	14.4%	1.0%	10.0%	3.0%	17.1%
**Used online resources**						
Daily	68.2%	46.7%	84.2%	36.6%	79.8%	40.3%
Weekly	24.5%	43.1%	10.4%	30.6%	9.6%	29.5%
Monthly	0.5%	7.2%	0.0%	0.0%	3.0%	17.2%
This resource was not available	5.2%	22.3%	5.0%	21.7%	4.5%	20.9%
Resource available, but not used	1.6%	12.4%	0.0%	0.0%	3.0%	17.2%

### Explanatory factors related to performance on the endline examination

Using linear regression with examination scores as the dependent variable, factors predictive of higher endline test scores were gender (female, +2.1% points, P< 0.01); younger age (below median age, +5.6% points, P< 0.001); and higher baseline test scores (+0.5% points for every correct answer on the baseline exam, P < 0.001). By contrast, group allocation and years of experience working as a CBPA had no association with endline scores. In our second model, limited to Group 2 and 3 participants, higher participation rates were associated with higher test scores (above average participation, +1.5 points, P = 0.14); when limiting to Group 3 participants, and also adjusting for correct SMS answer rates, more accurate responders had significantly higher endline exam results (above average accuracy, +3.2 points, P = 0.03).

## Discussion

A common criticism of mHealth interventions is that many appear promising, but few are tested in rigorous clinical trials, thereby precluding reliable determinations of effectiveness.[13, 14] In the mCME project, our results were unambiguous: our intervention, while technically feasible and well accepted by the CBPAs, did not achieve the goal of improving medical knowledge. Neither the daily bullet points nor the daily SMS questions improved performance on a tightly controlled standardized examination, nor incurred the secondary benefits of improved self-efficacy or job satisfaction.

In considering the possible reasons for this result, we dispensed with a number of problems common to randomized trials. While we did not achieve our target sample size at enrollment, loss to follow up was lower than anticipated, so statistical power was adequate. The exam and other baseline scores were nearly identical across the three groups at baseline, indicating that the randomization process yielded balance across the three groups with no indication of biased allocation.

In terms of potential bias in exam scores, we used a robust evaluation procedure to minimize cheating. Participants in all three groups often worked in the same clinics, and might have worked collaboratively to respond to Group 3 SMS questions. A possible result of that would have been an increase in scores across all three groups due to cross-contamination between intervention groups. However, the scores did not increase in any of the groups, so this concern remains hypothetical. Participation rates were high throughout the project among Group 2 and 3 participants, and positive attitudes towards the intervention were nearly universal. And the participants deemed the SMS content to be highly relevant. So lack of interest, low rates of participation, or a mismatch between the themes addressed and the daily work of CBPAs were not to blame.

We can also exclude technical barriers as an explanation since over 98% of the SMS messages were sent as scheduled. Similarly, the rate of Group 3 non-conforming answers received was low and improved early in the study, suggesting that the feedback loop (whereby an answer (correct or not) with a non-conforming format triggered a response with the correct format) was highly effective.

Several lines of evidence provide insight into what may actually have produced this result. First, among Group 3 participants, the proportion answering the daily questions correctly did not improve over the 6-month intervention period. It is true that the questions were not clustered thematically, meaning that a diabetes question on Tuesday could be followed by a surgery question on Wednesday, and a question about gynecology on Thursday. Nonetheless, frequent wrong answers did not lead the CBPAs to adopt better strategies for finding correct answers over time. And while consultation with colleagues and the use of the internet to find answers were common, neither are reliable sources for technical medical information. By contrast, CBPAs were far less likely to consult medical texts—a more reliable source of accurate information—even though most reported having access to textbooks. And lastly, the time CBPAs spent on weekly self-study was minimal and did not increase from baseline to endline.

It is helpful to consider our findings against the conceptual model we used to predict how the intervention might work (**Fig 1**). We considered this model to include a weak pathway (the SMS content) and a strong pathway (lateral learning). SMS messages are very short (<160 characters) and so cannot convey enough information to be the medium of content delivery *per se* (the weak pathway). Rather the intervention was intended to stimulate self-study on the same themes presented in the SMS message (the strong pathway). That is, we envisioned that a CBPA who received a question about stomach cancer, for instance, would be motivated to learn more about stomach cancer by consulting other sources (such as a textbook)—that is, engage in lateral learning. Yet, we learned at the endline evaluation that many CBPAs copied down the daily SMS into personal ‘mini texts’, studied, and memorized them, and often shared these with colleagues. Since the endline examination covered the same thematic areas as the texts, but by design did not repeat the same questions as the SMS messages, this strategy of memorizing answers to the messages was wasted effort.

In retrospect, we believe that a limitation of our study design was that we did not provide the CBPAs with reference materials linked to the SMS or with explicit instructions about how the SMS should help guide self-study behaviors. This raises a relevant question: if such resources were provided and those assumptions made explicit as instructions, could SMS messages serve as an effective stimulus to promote lateral learning? Our own work using SMS reminders to promote HIV medication adherence, along with other studies, suggest strongly that SMS can be a potent stimulus for behavioral change.[15–17]

Our study had several other limitations, some of which have been discussed above. First, it was often the case that CBPAs randomized to different groups worked side by side in the same clinics, and had ample opportunity to share the SMS messages. On one level, this is desirable, since our goal is to encourage dissemination of knowledge. But within the context of an RCT, such cross-contamination could drive the apparent effectiveness of the intervention towards the null. In the current case this concern remains hypothetical since the endline examination scores did not improve in any of the groups. A second limitation was that the BIAJS and CSE tools had only been validated in English speaking cohorts, and we did not have resources to re-validate these secondary endpoint tools for a Vietnamese population. A third limitation was that the SMS messages were delivered in random order rather than being clustered into thematic areas. It is possible that such clustering could have rendered the information more impactful, and this approach will be built into future iterations of the mCME intervention.

With these considerations in mind, our group has been funded to conduct a follow-on study of the mCME concept, but focusing on HIV/AIDS clinical care knowledge among Vietnamese HIV clinicians. Building on experiences in this first mCME study, the HIV/AIDS study will utilize SMS as a tool to link individuals to more detailed information on the same topic as in the message, and to encourage use of online accredited CME materials (of which many exist in Vietnam but are currently underutilized). Such materials designed specifically for CBPAs do not exist in Vietnam currently, though developing them is an ongoing focus of the MOH.

While there are myriad examples of pilot or feasibility projects evaluating SMS for distance learning,[18–22] we could only identify three published efficacy studies. Each of these was focused very narrowly on specific medical problems rather than supporting a broad base of medical knowledge as in the mCME Project. The first used SMS-based learning among family practice medical residents studying musculoskeletal medicine.[23] While limited by high and differential attrition, intervention and control groups both improved relative to baseline scores. However, SMS conferred no additional benefit over traditional self-study. This is relevant because it shows that lateral learning can be achieved among certain user groups, but leaves open the operational question of how to use SMS to enhance lateral learning. The second study, conducted among Chinese primary care physicians, compared the effectiveness of SMS messages vs. in-person CME training on the appropriate management of viral respiratory tract infections. The study found a significant improvement in medical knowledge among the SMS recipients. This supports SMS as a tool for distance learning, at least when focused narrowly.[24] A third very small pilot study compared textbooks vs. SMS on knowledge of breast cancer screening practices. This also found a small improvement among the SMS participants at endline. However, the pre-intervention and post-intervention examinations were identical making it hard to parse the effects of memory of the prior exam from the SMS intervention *per se*.[18] Collectively, these studies highlight the feasibility and possible utility of SMS to guide distance learning, but provide little insight into how SMS could be used to support a broad range of medical knowledge.

In summary, the strategy of using SMS text messaging was unsuccessful at improving medical knowledge, and had no impact on self-efficacy or job satisfaction among Vietnamese CBPAs. We hypothesize that this was because the mCME intervention did not achieve the requisite goal of stimulating lateral learning. While disappointing, the intervention was well received by the participants, and technical barriers were minimal and surmountable. Thus, we concluded that the mCME approach was feasible and acceptable. The implications of this from the perspectives of practice and research remain significant. While the study did not yield the desired result, the research was highly informative and encouraging in other ways. Not least of which is the fact that the CBPAs wanted to engage in CME, found the mCME intervention a convenient and fun way to do this, and were highly motivated by the process. This is not merely a matter of claiming that the glass is half full because many of the adjustments to the intervention that emerged in the qualitative data (thematic clustering of material; providing linkages to technical resources to support lateral learning; creating links to other CME activities) can and will be incorporated into future versions of the current intervention. In short, we learned a great deal from this investigation and do not feel that the concept of SMS supported distance learning should be abandoned. Quite the opposite: tour findings combined with evidence that using SMS can change behavior, supports optimism that refinements to this strategy may yet be successful. The challenge will be to better couple the stimulus (SMS messages) to long-term behavior change (self-study). We believe that a potential key to doing just this might be to directly link the stimulus with appropriately vetted learning materials, whether paper or digital. Our team will evaluate this approach in our next study among Vietnamese HIV/AIDS doctors.

## Supporting Information

S1 Checklist Consort Checklist. (DOC)

S1 Data Final data sets. (7Z)

S1 Protocol Final protocol in effect prior to subject enrollment. (DOCX)
