# Supplementary material for: The mCME Project: A Randomized Controlled Trial of an SMS-Based Continuing Medical Education Intervention for Improving Medical Knowledge among Vietnamese Community Based Physicians’ Assistants
Source: PLoS One. 2016 Nov 18;11(11):e0166293. doi: 10.1371/journal.pone.0166293 (PMC5115715; doi:10.1371/journal.pone.0166293)
Supplement: S1 Protocol — (DOCX) [file pone.0166293.s003.docx]

**1. Introduction**

**1.1 Background and objectives**

In the mCME project, we propose to test the effectiveness of a mobile phone-based CME delivery strategy among Vietnamese community-based physician assistants (CBPAs), a cadre of CHW mandated to provide primary health care to rural and disadvantaged populations. Effectiveness will be assessed using a three-armed randomized controlled trial involving 660 CBPAs. **Group 1** will serve as control, with CME delivered solely through the existing system of user-directed self-study and periodic, government sponsored in-service trainings; training for Group 2 and 3 subjects will be supplemented through either of two versions of the mCME intervention. **Group 2** subjects will receive daily SMS text messages spanning various domains of their professional duties. **Group 3** subjects will receive a daily SMS text message presented as a multiple-choice question, to which the CBPAs key in a response on their phone. This enables us to contrast the effectiveness of mCME content delivered passively (Group 2) or interactively (Group 3). At baseline and at the end of six months, all subjects will undergo a standardized exam. We hypothesize that CHWs who received the SMS text reminders will outscore CHWs who did not receive reminders, and that CBPAs assigned to the ‘interactive’ mobile CME model (Group 3) will be more engaged in this activity, and outscore those assigned to the ‘passive’ mobile CME model (Group 2). We further hypothesize that the mCME strategy will be cost-effective, and incur the secondary benefits of improved job satisfaction and self-efficacy among the participants.

**1.2 Specific aims**

The study will assess the following primary and secondary specific objectives:

| **PSA1** – To assess the effect mobile CME vs. no CME (Groups 2+3 vs. 1) |
| --- |
| **PSA2** – To compare the effect of ‘passive’ vs. ‘active’ mobile CME (Group 3 vs. 2) |
| **SSA1** – To assess the effect of active mobile CME vs. existing standard (Group 3 vs. 1) |
| **SSA2** – To assess the effect of passive mobile CME vs. existing standard (Group 2 vs. 1) |
| **SSA3** – To compare levels of self-efficacy and job satisfaction across the 3 groups (all groups) |
| **SSA4** – To qualitatively assess the acceptability of the mCME intervention (all groups) |
| **SSA5** – To determine the cost effectiveness of the mobile CME intervention (all groups) |

**1.3 Study team, key personnel**

The key study members include the following individuals

1. Dr. Christopher Gill, MD MS, Associate Professor of Global Health, BUSPH. Role: Principle Investigator
2. Dr. Lora Sabin, PhD MA, Associate Professor of Global Health, BUSPH. Role: Co-Investigator
3. Marion McNabb, MPH, Technical advisor, Pathfinder International. Role: Co-investigator, primary liason with VN field team.
4. Mr. Bao Ngoc Le, MA, MS, Country Representative in Vietnam, Pathfinder International. Role: Co-investigator, lead for VN field team; liaison with VN MOH and public health agencies.

**2. Methods**

**2.1 Recruitment and Eligibility**

Study subjects are individuals are licensed graduates of an accredited CBPA training program who are registered by the VN MOH. It is hoped that the cohort an be recruited from the single province Nghe An. If necessary, enrollment can be expanded to additional provinces, or by sequential cohorts within Nghe An. This will be determined as the study progresses. In all cases, the process of reaching out to and enrolling CBPAs will be coordinated with the VN MOH, which maintains centralized registration data on all the CBPAs licensed to work in the country.

Potential subjects will be included provided that they meet all of the following inclusion and exclusion criteria:

| *Inclusion* | *Exclusion* |
| --- | --- |
| 1. Licensed graduate from a CBPA training program | 1. Unwilling to sign informed consent |
| 1. Possesses their own cell phone | 1. Lives/operates in an area without cellular coverage |
| 1. Phone able to send/receive text messages | 1. Unwilling to adhere to study procedures |
| 1. Aged ≥18 years |  |

**2.2 Study Procedures**

**2.2.1 Allocation of subject IDs**

Subjects will be assigned a unique study ID no. once they have signed consent. These will be provided sequentially as subjects enroll.

A key will be created to link subject names to the assigned ID numbers. At the conclusion of the study this key will be destroyed.

**2.2.2 Randomization**

Following the examination, subjects will be allocated to 1 of 3 groups using a process of restricted randomization designed to try and reach parity in the #s of CBPAs in each group. Ideally, 220 subjects will be randomized to each group. The results of the randomization will be provided to each subject at the time of randomization. Randomization will be based on a pre-defined randomization list linked to subject IDs.

**2.3 Data collection**

Once the required numbers of CBPAs have been consented and randomized, subjects will be scheduled for their baseline assessment. This will occur centrally, either in one location or at several geographically located areas roughly in parallel (these need not occur on precisely the same day, but we aim to have these all done within the same week). Each examination booklet will be pre-printed at the top of each page with a given subject’s unique study ID no. to match the booklet to the randomization schema. At this point, the study ID no. will be linked to each subject’s cell phone number to allow the SMS reminders.

The baseline assessment will include the following elements:

**Part A. Self entered demographic information.** This includes information regarding age, sex, marital status and # of dependents for the CBPA; educational history (general education and medical education specifically); date of graduation from CBPA training; name of training college; when most recent re-training occurred; total # of govt. sponsored retraining sessions attended in the last 2 years; location of current posting; years worked at current post; description of posting site (rural, small town, urban). This should require about 5 minutes to complete. Part A will only be administered at the baseline evaluation.

**Part B. Self entered responses to job satisfaction and self-efficacy surveys.** Each of these will require about 2-3 minutes to complete. This will be administered at the baseline and endline evaluations.

**Part C. Professional knowledge assessment test**. This is the examination that measures each CBPA’s knowledge of key concepts related to their clinical training, and will be based on the standard textbook employed in their primary training. The exam will consist of 100 items in multiple-choice format. Students will have 2.5 hours to complete the exam. This will be administered at the baseline and endline evaluations.

Four versions of the test will be created, each covering similar domains of relevant knowledge based on the CBPA text, but using different questions. Versions 1.1 and 1.2 will include identical questions, but the order of the questions will be scrambled; Similarly versions 2.1 and 2.2 will include identical questions (and though covering the same thematic areas as version 1 will be all new questions), but the order of the questions in 2.1 and 2.2 will be scrambled. At the end of six months, when the CBPAs repeat the exam, each individual will use the alternate version to the one that they took initially, i.e., a CBPA who took version 1.1 or 1.2 at baseline will be administered 2.1 or 2.2 at endline; a CBPA who took version 2.1 or 2.2 at baseline will take 1.1 or 1.2 at endline.

This serves two objectives. First it helps minimize the risk of cheating during exams, since adjacent neighbors sitting in an exam hall are unlikely to be taking the same version of the exam as a given tester (so copying answers will not be possible). Second, it reduces the possibility that improvements on the test are simply because they remembered questions from the previous testing date.

Additional strategies to reduce the risk of cheating are:

1. All subjects must present an official photo ID in order to receive their test book (e.g., driver’s license, national ID card, passport)
2. The examinations will be proctored.

Scores in the exam will be converted to a simple numerical score on a 0-100 scale.

**2.4 The intervention**

Following the baseline evaluation, CBPAs will return and resume their regular professional activities. Depending on their randomization group, subjects will either receive no SMS reminders (Group 1 – controls); will receive a daily reminder in the form of a single bullet point fact relating to some aspect of the professional duties (Group 2 – intervention, passive); or will receive a daily multiple choice question, covering the same thematic areas as with group 2 (Group 3 - intervention, interactive).

For group 2/3 subjects, the SMS reminders will typically be short enough to nest within the size limits of a single message. However, longer messages can be delivered over 2 or more consecutive SMS messages if needed.

Data will be collated based on the replies back from Group 2 and 3 subjects and assessed collectively in terms of response rates within and across groups 2 and 3; time from when messages sent to when answers received (on average and by day of the week); and, for group 3 subjects, proportion of correct/incorrect responses.

Group 2 subjects are instructed simply to ‘reply’ to the daily message by typing any key on their touch pad and hitting the send button. This is to acknowledge that the message was received.

Group 3 subjects are instructed to key in an answer to the question. The questions will be presented usually as 4 option answers, and so the subject would enter in their answer among the numbers on the phone touch pad, and then hit the send button.

At the end of six months, or specifically after 180 days have elapsed, allowing for 180 text reminders to be sent, the second assessment will be convened. Again, subjects will present to take this assessment as a proctored examination as described above. As opposed to the baseline evaluation, baseline demographic data is not collected, but only items B and C as described above.

**2.5 Development of SMS content and exam content**

These will be based on the standard textbook used for training CBPAs in VN. Key thematic areas for focus will be identified by the key study team members, in consultation with the VN MOH and/or public health agencies. Daily SMS reminders and MCQs will be developed jointly by students at Boston University School of Public Health and public health students in VN. Translation will be conducted into Vietnamese by local collaborators. Similarly, the Versions 1.0 and 2.0 of the exam will be generated based on the standard textbook, and translated locally into Vietnamese.

**2.6 Qualitative investigations**

Following the second assessment, within groups 2 and 3 we will purposefully invite 80 individuals to participate in in-depth interviews (IDIs) and focus group discussions (FGDs) of 6-8 participants, to be held the same day as the endline test. These will be audio recorded and translated into English for analysis.

These subjects will be provided lunch.

The FGDs and IDIs will assess the following general areas:

1). CBPAs attitudes regarding continuing medical education opportunities in Vietnam

2). Pros and Cons of the current system for supporting medical knowledge

3). Their impressions/opinions/likes/dislikes regarding the daily text messages, including whether they found them useful, intrusive, annoying, and whether the information that was presented was appropriate in content, format, relevance and complexity.

At this point, the data collection phase of the study is complete.

**3. Analysis**

**3.1 Analytic methods**

| **Specific Aim** | **Group comparisons** | **Outcome** | **Analytic approach** |
| --- | --- | --- | --- |
| PSA1 – To assess the effect mobile CME vs. no CME | Groups (3+2) vs. 1 | CBPA test performance at the end of six months | **Primary strategy**   - Independent T-tests or Mann-Whitney U tests - Linear regression (mean scores) - Logistic regression (passing/non-passing)   **Secondary strategy**   - Paired T-tests or Mann-Whitney U test for a matched pair analysis of pre-post test results within each group |
| PSA2 – To compare the effect of passive vs. active mobile CME | Group 3 vs. 2 | CBPA test performance at the end of six months |  |
| SSA1 – To assess the effect of active mobile CME vs. existing standard | Group 3 vs. 1 | CBPA test performance at the end of six months |  |
| SSA2 – To assess the effect of passive mobile CME vs. existing standard | Group 2 vs. 1 | CBPA test performance at the end of six months |  |
| SSA3 – To assess and compare levels of self-efficacy and job satisfaction at end of six months | Groups 3 vs. 2 vs. 1 | - CSE[^47^](#_ENREF_47) scale - BIAJS[^46^](#_ENREF_46) scale |  |
| SSA4 – To determine the acceptability of mCME | Groups 2 and 3 only | Qualitative data from Focus Group Discussions | - Domain analysis |
| SSA5 – To determine the cost effectiveness of mobile CME | Groups 3 vs. 2 vs. 1 | Marginal costs in US Dollars | - Incremental Cost Effectiveness Analysis |

All subject data, linked to study ID Nos., will be collected on paper case report forms. Section A data will be converted to digital format through dual data entry, with reconciliation against the original paper source document between the two data entries. Section B and C data will be entered on bubble sheets and will either be entered manually via dual data entry, or automatically by digitally scanning the sheets.

Results from sections B and C will be converted to raw scores with medians, IQRs, means and standard deviations. Comparisons of mean scores will be made across each of the three groups using T tests and linear regression or other tests as indicated (see table above). In addition, the proportion of subjects scoring 85% or higher on section C will be compared using chi square tests and logistic regression or other tests, as indicated.

Analysis of the qualitative data will use the abstracted notes taken during the FGDs and IDIs to identify common themes across the different domains included in the discussion guides. Data will be extracted and analyzed using nVivo software.

Cost effectiveness will be assessed using the standard economic approach as follows:

CEA =(Cx-Cy)/(S*x*-S*y*)

Where CEA is cost effectiveness analysis, C is total cost of the relevant interventions, x and y refer to the two groups being compared (in accordance with the specific aims), and S is the score on the standardized exam, assessed in two ways: 1) cost of each additional student scoring above a specified threshold score on the exam (e.g., 90%) and 2) cost for a 5 point increment in mean test scores. Costs will include all those related to implementing the intervention (development of questions and potential responses, SMS delivery) but exclude research costs. We will express costs in real, discounted local currency and US dollars. Additionally, given relatively high start-up costs, we will estimate cost effectiveness over a future 10-year timeframe to provide policy-relevant programmatic data.

**3.2 Data storage**

Once primary data collection is complete (i.e., immediately after the endline assessment at six months in the study, and once all FGDs and IDIs are complete), and the database has been cleaned and locked, the subject ID key will be destroyed.

Paper records will be warehoused in a secure, locked space at the study management center in Vietnam and retained for at least 5 years. Digital records will be warehoused at Boston University.

**4. Study timeline**

The estimated timeline for the project is summarized below. Note that this table assumes a Jan to December cycle, whereas in reality these would likely be relative times based on the actual study start date.

| **Key Milestones** | **Y1**  **Q1** | **Q2** | **Q3** | **Q4** | **Y2**  **Q1** | **Q2** | **Q3** | **Q4** |
| --- | --- | --- | --- | --- | --- | --- | --- | --- |
| Finalize protocol/tools | X |  |  |  |  |  |  |  |
| IRB approvals received | X |  |  |  |  |  |  |  |
| Group 2/3 SMS materials developed |  | X |  |  |  |  |  |  |
| SMS system tested/validated |  |  | X |  |  |  |  |  |
| Staff Training completed |  |  | X |  |  |  |  |  |
| Baseline CBPA exam administered |  |  |  | X |  |  |  |  |
| Period of intervention |  |  |  | X | X | X |  |  |
| Endline CBPA exam administered |  |  |  |  |  | X |  |  |
| Data analysis workshop |  |  |  |  |  |  | X |  |
| Abstracts/Papers written |  |  |  |  |  |  |  | X |
